# An Unusual Presentation of Lupus Vulgaris and the Practical Usefulness of Dermatoscopy 

**DOI:** 10.1155/2018/1036162

**Published:** 2018-11-14

**Authors:** Grigorios Theodosiou, Marina Papageorgiou, Ioanna Mandekou-Lefaki

**Affiliations:** ^1^Specialist in Dermatology-Venereology, Department of Dermatology, Skåne University Hospital, Jan Waldenströms gata 16, 20502 Malmö, Sweden; ^2^Specialist in Dermatology-Venereology, State Clinic of Dermatology, Hospital for Skin and Venereal Diseases, Delfon 124, 54643, Thessaloniki, Greece; ^3^Specialist in Dermatology-Venereology, Chief of State Clinic of Dermatology, Hospital for Skin and Venereal Diseases, Delfon 124, 54643 Thessaloniki, Greece

## Abstract

We report a case report of lupus vulgaris (LV) presented on the extremities of an 80-year-old man. He was misdiagnosed as having psoriasis and referred to our department for a second-opinion evaluation. The discrepancy between the clinical manifestation which was misleading and the dermatoscopic findings raised the suspicion of an underlying granulomatous disease and we proceeded to skin biopsy. The histopathologic examination set the diagnosis of LV.

## 1. Introduction

Lupus vulgaris (LV) is the most common form of cutaneous reinfection with M. tuberculosis. It occurs predominantly in young adults. LV affects primarily the head and neck region. LV in regions other than the head and neck can pose diagnostic difficulties. We report a case of LV localized on the extremities of an elder patient and we stress the role of dermatoscopic examination.

## 2. Case Report

An 80-year-old man of Caucasian origin diagnosed with late-onset plaque psoriasis was referred to our Department for a “second-opinion” evaluation and eventually administration of systemic treatment. All lesions appeared at least 2 years before the dermatologic assessment at our department. The patient had received topical therapy with fixed combination of calcipotriol/betamethasone once daily for 3 months and subsequently with clobetasol for around 4 months once daily without any response. The family history of the patient was negative for psoriasis or other chronic skin diseases. Our patient had no previous dermatologic history and was medicating for hypertension, GERD, and hyperlipidemia.

**Figure 1:**
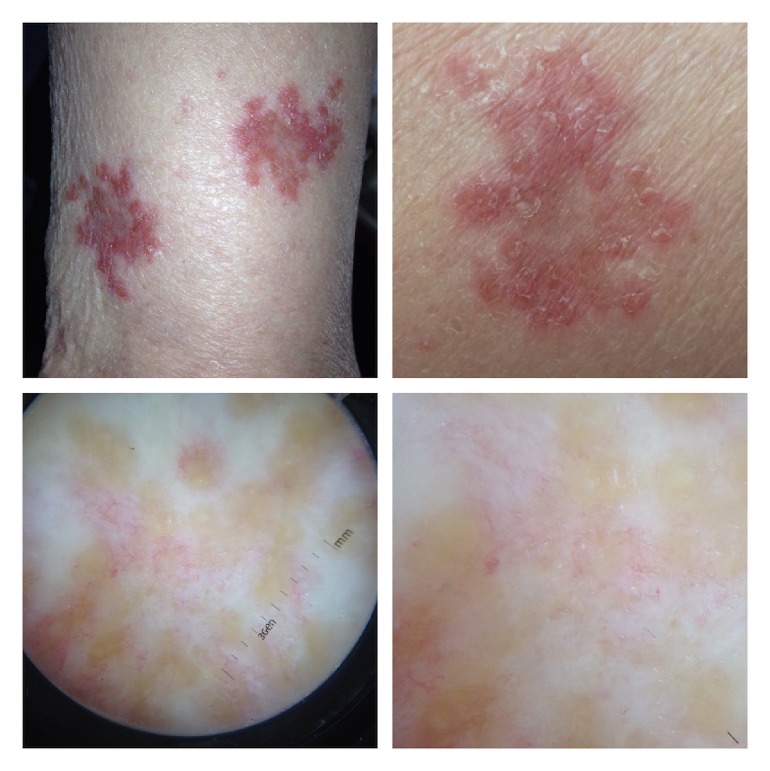
(a, b) Clinical presentation: scaly well-demarcated plaques on the extremities of the patient. (c, d) Dermatoscopy: fine telangiectasias on yellow to golden background.

The clinical examination revealed well-demarcated erythematosquamous plaques with irregular shapes on the upper and the low extremities of the patient (Figures 1(a) and 1(b)). The clinical differential diagnosis included psoriasis, lupus erythematosus, tinea incognita, mycosis fungoides as well as leprae, leishmaniasis, sarcoidosis, and tuberculosis.

The dermatoscopic evaluation of the lesions revealed yellow-orange clods and focused, fine horizontal telangiectasias (Figures 1(c) and 1(d)). These findings are repetitively reported as suggestive for granulomatous skin diseases such as sarcoidosis, tuberculosis, and granuloma annulare. The correlation of the clinical and dermatoscopic features of the lesion suggested a diagnosis of a granulomatous disease such as cutaneous tuberculosis or sarcoidosis.

Biopsy of a representative lesion was performed and the histological examination revealed the presence of tuberculoid granulomas accompanied by caseation necrosis. The tuberculin skin test was found positive. PCR for M. Tuberculosis DNA done on a tissue sample was positive. The clinical, dermatoscopic, and microscopic features were consistent with the diagnosis of lupus vulgaris. The screening for an extracutaneous focus of TB was negative. Our patient was treated with a 2-month course of isoniazide 5 mg/kg/d, rifampicin 10 mg/kg/d, pyrazinamide 35 mg/kg/d, and ethambutol 20 mg/d followed by rifampicin and isoniazid for a further 4 months. Complete resolution of the rash with minimal residual scarring was observed in follow-up examination at the end of the treatment.

## 3. Discussion

Lupus vulgaris (LV) is a rare, chronic, progressive form of tuberculosis caused by continuous spread from an underlying focus of infection or by hematogenous or lymphatic spread [1, 2].

LV typically is characterized by red-brown papules, which coalesce to form a well-demarcated scaly, asymptomatic plaque. The plaque gradually expands by development of new papules at the periphery. When blanched by diascopic pressure, the lesions reveal a pale brownish-yellow or “apple-jelly” color [3–5].

Histologically, classic tubercles are the hallmark of LV. Caseation within the tubercles is seen in about half the cases and is rarely marked. PCR still lacks the sensitivity and specificity to diagnose paucibacillary forms of cutaneous TB [6].

Dermatoscopy, in addition to its well-documented value in evaluation of skin tumors, is continuously gaining appreciation also in the field of general dermatology [7]. Fine focused telangiectasias on a yellow to golden background have been described as the typical dermatoscopic findings of LV and are correlated with the apple-jelly sign [8, 9].

In our case the dermatoscopic examination of all lesions revealed yellow to orange areas intersected by fine horizontal telangiectasia. The dermatoscopic findings led us to consider granulomatous skin diseases in the differential diagnosis and to conduct a skin biopsy. Further confirmation of our observation by a larger series of cases is required.
